# Cardiovascular Risk Stratification in Diabetic Retinopathy via Atherosclerotic Pathway in COVID-19/Non-COVID-19 Frameworks Using Artificial Intelligence Paradigm: A Narrative Review

**DOI:** 10.3390/diagnostics12051234

**Published:** 2022-05-14

**Authors:** Smiksha Munjral, Mahesh Maindarkar, Puneet Ahluwalia, Anudeep Puvvula, Ankush Jamthikar, Tanay Jujaray, Neha Suri, Sudip Paul, Rajesh Pathak, Luca Saba, Renoh Johnson Chalakkal, Suneet Gupta, Gavino Faa, Inder M. Singh, Paramjit S. Chadha, Monika Turk, Amer M. Johri, Narendra N. Khanna, Klaudija Viskovic, Sophie Mavrogeni, John R. Laird, Gyan Pareek, Martin Miner, David W. Sobel, Antonella Balestrieri, Petros P. Sfikakis, George Tsoulfas, Athanasios Protogerou, Durga Prasanna Misra, Vikas Agarwal, George D. Kitas, Raghu Kolluri, Jagjit Teji, Mustafa Al-Maini, Surinder K. Dhanjil, Meyypan Sockalingam, Ajit Saxena, Aditya Sharma, Vijay Rathore, Mostafa Fatemi, Azra Alizad, Vijay Viswanathan, Padukode R. Krishnan, Tomaz Omerzu, Subbaram Naidu, Andrew Nicolaides, Mostafa M. Fouda, Jasjit S. Suri

**Affiliations:** 1 Stroke Monitoring and Diagnostic Division, AtheroPoint™, Roseville, CA 95661, USA; munjralsmiksha@gmail.com (S.M.); mahesh.nehu.333@gmail.com (M.M.); dr.anudeeppuvvula@gmail.com (A.P.); ankush.jamthikar1992@gmail.com (A.J.); tjujaray@gmail.com (T.J.); drindersingh1@gmail.com (I.M.S.); pomchadha@gmail.com (P.S.C.); surinderdhanjil@gmail.com (S.K.D.); 2 Department of Biomedical Engineering, North Eastern Hill University, Shillong 793022, India; sudip.paul.bhu@gmail.com; 3 Max Institute of Cancer Care, Max Super Specialty Hospital, New Delhi 110017, India; puneet1923@gmail.com; 4 Annu’s Hospitals for Skin and Diabetes, Nellore 524101, India; 5 Department of Molecular, Cell and Developmental Biology, University of California, Santa Cruz, CA 95616, USA; 6 Mira Loma High School, Sacramento, CA 95821, USA; nsuri574@gmail.com; 7 Department of Computer Science Engineering, Rawatpura Sarkar University, Raipur 492015, India; drrkpathak20@gmail.com; 8 Department of Radiology, Azienda Ospedaliero Universitaria, 40138 Cagliari, Italy; lucasabamd@gmail.com (L.S.); antonellabalestrieri@hotmail.com (A.B.); 9 oDocs Eye Care Research Laboratory, Dunedin 9013, New Zealand; renoh.chalakkal@auckland.ac.nz; 10 CSE Department, Bennett University, Greater Noida 201310, India; suneet.gupta@bennett.edu.in; 11 Department of Pathology, Azienda Ospedaliero Universitaria, 09124 Cagliari, Italy; gavinofaa@gmail.com; 12 The Hanse-Wissenschaftskolleg Institute for Advanced Study, 27753 Delmenhorst, Germany; monika.turk84@gmail.com; 13 Department of Medicine, Division of Cardiology, Queen’s University, Kingston, ON K7L 3N6, Canada; johria@queensu.ca; 14 Department of Cardiology, Indraprastha APOLLO Hospitals, New Delhi 110001, India; drnnkhanna@gmail.com (N.N.K.); ajitsaxena@hotmail.com (A.S.); 15 Department of Radiology and Ultrasound, University Hospital for Infectious Diseases, 10 000 Zagreb, Croatia; klaudija.viskovic@bfm.hr; 16 Cardiology Clinic, Onassis Cardiac Surgery Centre, 17674 Athens, Greece; soma13@otenet.gr; 17 Heart and Vascular Institute, Adventist Health St. Helena, St. Helena, CA 94574, USA; lairdjr@ah.org; 18 Minimally Invasive Urology Institute, Brown University, Providence, RI 02912, USA; gyan_pareek@brown.edu; 19 Men’s Health Centre, Miriam Hospital Providence, Providence, RI 02906, USA; martin_miner@brown.edu; 20 Rheumatology Unit, National Kapodistrian University of Athens, 15772 Athens, Greece; dwsobel@gmail.com (D.W.S.); psfikakis@med.uoa.gr (P.P.S.); 21 Department of Surgery, Aristoteleion University of Thessaloniki, 54124 Thessaloniki, Greece; tsoulfasg@gmail.com; 22 Cardiovascular Prevention and Research Unit, Department of Pathophysiology, National & Kapodistrian University of Athens, 15772 Athens, Greece; aprotog@med.uoa.gr; 23 Department of Immunology, Sanjay Gandhi Postgraduate Institute of Medical Sciences, Lucknow 226014, India; durgapmisra@gmail.com (D.P.M.); vikasagr@yahoo.com (V.A.); 24 Academic Affairs, Dudley Group NHS Foundation Trust, Dudley DY1 2HQ, UK; george.kitas@nhs.net; 25 Arthritis Research UK Epidemiology Unit, Manchester University, Manchester M13 9PL, UK; 26 OhioHealth Heart and Vascular, Columbus, OH 43214, USA; kolluri.raghu@gmail.com; 27 Ann and Robert H. Lurie Children’s Hospital of Chicago, Chicago, IL 60611, USA; jteji@mercy-chicago.org; 28 Allergy, Clinical Immunology and Rheumatology Institute, Toronto, ON L4Z 4C4, Canada; almaini@hotmail.com; 29 MV Centre of Diabetes, Chennai 600013, India; dr_chokku@yahoo.com; 30 Division of Cardiovascular Medicine, University of Virginia, Charlottesville, VA 22904, USA; as8ah@hscmail.mcc.virginia.edu; 31 Nephrology Department, Kaiser Permanente, Sacramento, CA 95119, USA; rajvivs888@gmail.com; 32 Department of Physiology & Biomedical Engineering, Mayo Clinic College of Medicine and Science, Rochester, MN 55905, USA; fatemi.mostafa@mayo.edu; 33 Department of Radiology, Mayo Clinic College of Medicine and Science, Rochester, MN 55905, USA; azra.alizad@gmail.com; 34 MV Hospital for Diabetes and Professor MVD Research Centre, Chennai 600013, India; drvijay@mvdiabetes.com; 35 Neurology Department, Fortis Hospital, Bangalore 560076, India; pudukode.krishnan@fortisheakthcare.com; 36 Department of Neurology, University Medical Centre Maribor, 1262 Maribor, Slovenia; omerzu.tomaz@gmail.com; 37 Electrical Engineering Department, University of Minnesota, Duluth, MN 55812, USA; dsnaidu@d.umn.edu; 38 Vascular Screening and Diagnostic Centre, University of Nicosia Medical School, Nicosia 2408, Cyprus; anicolaides1@gmail.com; 39 Department of Electrical and Computer Engineering, Idaho State University, Pocatello, ID 83209, USA; mfouda@ieee.org

**Keywords:** diabetic retinopathy, atherosclerosis, cardiovascular disease, surrogate biomarkers, artificial intelligence, risk stratification, risk assessment

## Abstract

Diabetes is one of the main causes of the rising cases of blindness in adults. This microvascular complication of diabetes is termed diabetic retinopathy (DR) and is associated with an expanding risk of cardiovascular events in diabetes patients. DR, in its various forms, is seen to be a powerful indicator of atherosclerosis. Further, the macrovascular complication of diabetes leads to coronary artery disease (CAD). Thus, the timely identification of cardiovascular disease (CVD) complications in DR patients is of utmost importance. Since CAD risk assessment is expensive for low-income countries, it is important to look for surrogate biomarkers for risk stratification of CVD in DR patients. Due to the common genetic makeup between the coronary and carotid arteries, low-cost, high-resolution imaging such as carotid B-mode ultrasound (US) can be used for arterial tissue characterization and risk stratification in DR patients. The advent of artificial intelligence (AI) techniques has facilitated the handling of large cohorts in a big data framework to identify atherosclerotic plaque features in arterial ultrasound. This enables timely CVD risk assessment and risk stratification of patients with DR. Thus, this review focuses on understanding the pathophysiology of DR, retinal and CAD imaging, the role of surrogate markers for CVD, and finally, the CVD risk stratification of DR patients. The review shows a step-by-step cyclic activity of how diabetes and atherosclerotic disease cause DR, leading to the worsening of CVD. We propose a solution to how AI can help in the identification of CVD risk. Lastly, we analyze the role of DR/CVD in the COVID-19 framework.

## 1. Introduction

The mortality of 17.9 million people every year in the world is due to cardiovascular diseases (CVD) [1]. Meanwhile, atherosclerosis is considered one of the main leading causes of CVD [2,3], and several other factors, such as prolonged diabetes mellitus (DM) and lifestyle factors, are attributed to it as well. Diabetes is a chronic disease and is responsible for 1.5 million deaths worldwide [4,5]. The number of people suffering from diabetes is rising every year. A 2018 consensus report suggested that 34.2 million people had diabetes, out of which 7.3 million remained undiagnosed [6]. The poor healthcare system in developing countries has led to a further worsening of the condition. There are currently 285 million people in the world who have diabetes mellitus (DM). By 2030, the number of people suffering from DM is expected to rise to 439 million [7]. Apart from being a leading factor in causing CVD, diabetes also doubles the threat of stroke [8,9]. Uncontrolled DM leads to blindness, renal failure, myocardial infarction, and lower limb amputation [10]. Specifically, it often leads to diabetic retinopathy (DR), a pathological condition affecting the vision. Globally, DR affects 93 million people [11,12]. Therefore, it is important to know the causes of diabetes that lead to the formation of DR and the role of retinal imaging in evaluating the stages of DR.

DM develops due to increased levels of blood sugar in our bodies, causing damage to blood vessels as time progresses [13,14]. This affects the tiny network of blood vessels in the eye [15]. Since the retina of the eye is a sensory membrane that requires a regular supply of blood [16], a person suffering from diabetes may experience vision loss after suffering damage to these blood vessels. This well-known condition is DR [17,18]. Furthermore, poor glycemic control, high cholesterol, microalbuminuria, smoking, and vasoconstriction from high blood pressure all contribute to some of the risk factors associated with DR [19,20,21].

Many investigations have found a link between DR and CVD [22,23]. DR is an indication of active and uncontrolled diabetes and thereby increases the risk of CVD [24,25]. They can also trigger inflammatory responses that can contribute to atherosclerosis also causes CAD and worsening CVD [3,24,25]. Obesity, hypertension, and hyperlipidemia are major risk factors for both DR and CVD [26]. Several studies link more severe Atherosclerotic Cardiovascular Disease (ASCVD) to more advanced DR stages [27,28,29,30]. Therefore, DR promotes CVD it is imperative to understand the association between DR and CVD to minimize heart attacks, cardiovascular events (CVE), and the risk of stroke [31,32].

Retinal imaging is a vital practice in DR investigation with increasing DR, alterations such as hard exudate development and hemorrhage development elevate the risk of CVD [33]. Using retinal imaging to track DR changes is critical to determining the severity of DR [34]. When assessing CVD risk, coronary imaging is recommended [35]. Furthermore, coronary artery imaging is required to observe plaque in CAD. Effective imaging methods for detecting coronary plaque include intravascular ultrasonography and optical coherence tomography [36]. There are various previous studies [22,26,37,38,39] that do not clearly explain the details, which includes (i) direct studies of DR-CVD using AI (ii) granular risk prediction using AI (iii) studies of bias in AI (iv) DR-CVD in a surrogate framework and (v) effect of COVID-19 on DR-CVD. Thus, there is a clear need for (i) reliable and automated carotid plaque risk assessment, (ii) CVD risk stratification, and (iii) early monitoring of atherosclerotic disease in DR patients. These three elements are critical in the detection of high-risk CVD in DR patients from worsening.

The healthcare industry has been revolutionized by Artificial Intelligence (AI). Many medical applications have utilized Machine Learning (ML) and Deep Learning (DL) algorithms [40,41]. AI-based solutions are data-driven, using database information to make judgments. It finds non-linear relationships between input predictors and cardiovascular outcomes [42]. ML-based algorithms may employ complicated, non-linear relationships among several input risk predictors (or attributes) at once, unlike existing statistical risk prediction models [42,43]. To create predictions, DL algorithms extract features directly from the input data, for example, carotid wall tissue characterization, picture segmentation, and CVD risk stratification [44]. The use of DL algorithms (CNNs) to extract features and then train/test an ML classifier to gain final classification has also been demonstrated [45,46]. Recently, retinal pictures have been used to predict coronary artery calcium scores and estimate CVD risk [47,48]. ML and DL-based algorithms have been used to predict diabetic retinopathy [49,50,51,52]. Therefore, it is possible by reducing the requirement for human intervention, AI-based solutions enable the examination of image-based retinal inputs [53]. Several carotid ultrasonography applications utilizing AI-based algorithms have shown promise [54,55]. Thus, these AI-based algorithms may be used to concurrently handle CVD and DR diseases in patient risk assessment.

**Figure 1:**
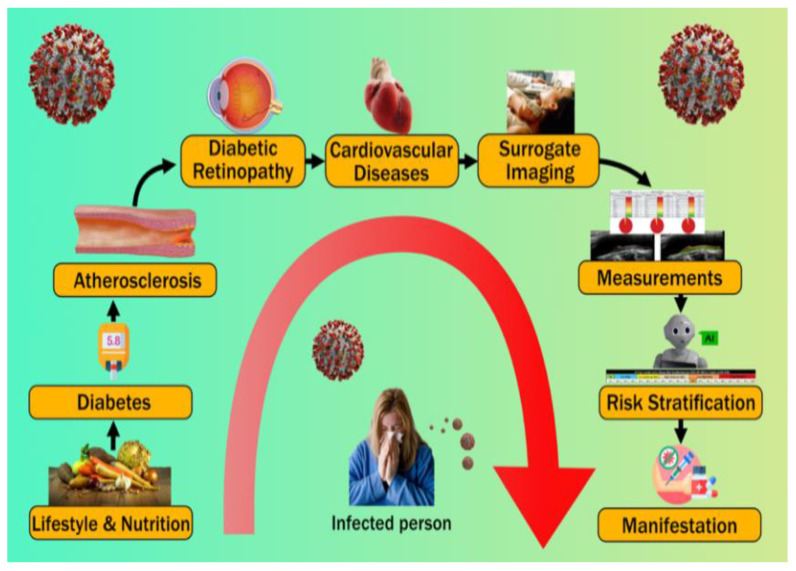
Pathophysiology cycle of DR and CVD.

This study focuses on the use of low-cost carotid ultrasound imaging to better understand the pathophysiology of diabetes, diabetes-related kidney disease, and cardiovascular disease. The use of surrogate imaging for CAD visualization also assists in the categorization of DR patients into suitable CVD risk groups [56,57]. Patients at high risk of developing diabetic complications can be identified using machine learning and deep learning techniques [58]. An overview of the disease in a COVID-19-influenced environment provides insight into the current issues for disease treatment and pathogenesis. Figure 1 illustrates the pathophysiology cycle of DR and CVD.

The main contributions of this study are as follows: (i) establishment of DR-CVD hypothesis (Section 3); (ii) carotid arterial imaging surrogate biomarker for DR-CVD framework (Section 4); (iii) establishment of AI-based CVD risk assessment (Section 5); (iv) workflow design for CVD risk assessment in presence of DR and COVID-19 (Section 6); (v) benchmarking our current CVD paradigm against previous studies (Section 7).

## 2. Search Strategy

**Figure 2:**
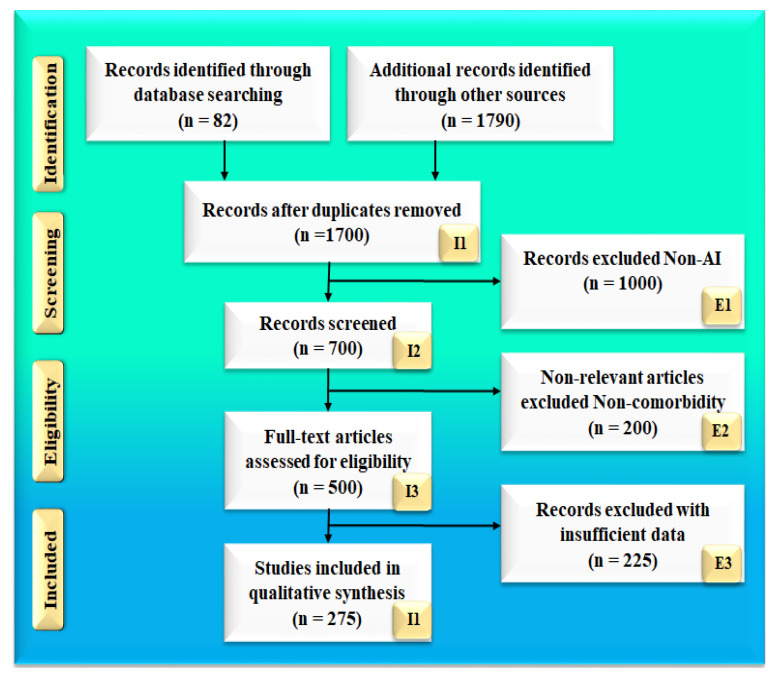
Search strategy based on the PRISMA model.

The search strategy follows the PRISMA model, which is shown in Figure 2. Two popular databases, PubMed and Google Scholar, were used to find and screen the relevant articles using keywords such as “diabetic retinopathy”, “diabetes”, “CVD”, “diabetic retinopathy and CVD”, “diabetic retinopathy and coronary artery disease”, “retinal imaging”, “carotid imaging”, “artificial intelligence”, “atherosclerotic plaque tissue classification and characterization”, “artificial intelligence”, “diabetic retinopathy, diabetes and COVID-19”, “atherosclerosis and COVID-19”. A total of 82 articles on PubMed and 1790 articles on Google scholar were identified. Advanced criteria, such as time and relevance, were applied to reduce the list to 500 articles. Out of which 198 articles were screened for inclusion in this review, and a final list was provided. The three exclusion criteria were as follows: (i) studies that were not connected to our objectives, (ii) articles that were not relevant, and (iii) research that had insufficient data in the studies. Thus, 270 studies were ultimately selected after the exclusion of 1000, 200, and 225 investigations (labeled with the letters E1, E2, and E3), for a total of 275 studies. The complete screening process is shown in Figure 2.

## 3. Diabetic Retinopathy

Diabetes is defined by abnormally high blood glucose levels (hyperglycemia) in the body [59]. This is primarily owing to the body’s poor production and/or usage of insulin [60]. If left unchecked, diabetes’ metabolic abnormalities can result in a plethora of acute and chronic consequences [61]. Chronic problems are further classified as microvascular or macrovascular. Neuropathy, diabetic retinopathy, and nephropathy are all examples of microvascular consequences. Ischemic heart disease, cerebrovascular illness, and peripheral vascular disease are all macrovascular consequences [62,63,64].

Among these, DR is the most recurrent and common complication [65,66,67]. It is characterized as a complex ocular manifestation of diabetes that leads to the alteration of various pathways affecting the retina [66,68,69]. The basic indicators of DR include loss of pericytes, microaneurysms, lipid deposits (exudates), neovascularization, thickening of the basement membrane, and breaking down of the blood–retinal barrier [68].

The Human eye is a vital organ that consists of the cornea, pupil, iris, retina, lens, and sclera [69].

**Figure 3:**
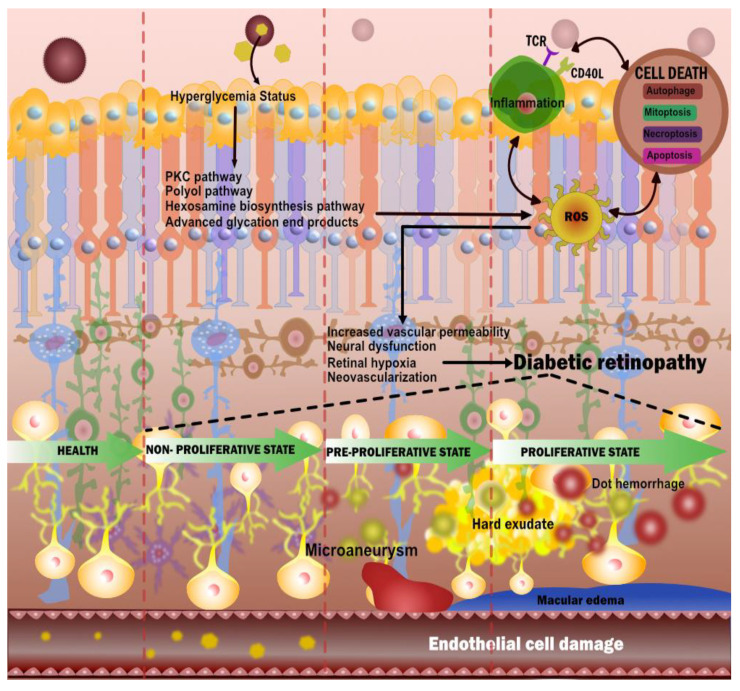
Pathophysiology of diabetic retinopathy.

The retina, which is a thin layer of highly metabolically active tissues positioned near the optic nerve, is one of these. On the interior, it encircles the rear of the eye [70,71]. The retina’s function is to receive light signals focused by the lens and convert them to neural impulses [72]. Additionally, it transmits these impulses to the brain for visual recognition. Hyperglycemia impairs the function of the retina’s vascular endothelial cells [73]. In general, the four major pathways involved in the prognosis of DR are (i) increased polyol pathway flux, (ii) activation of protein kinase C (PKC), (iii) increased advance glycation end products (AGE) formation, and (iv) polyol pathways (Figure 3) [74].

These pathways interchangeably cause oxidative stress and can lead to inflammation and apoptosis [75]. Further, neurovascular damage which includes neural dysfunction, retinal hypoxia, and increased vascular permeability can ultimately lead to DR [76]. Neovascularization leads to hemorrhage and fibrosis, which further leads to traction [77].

**Figure 4:**
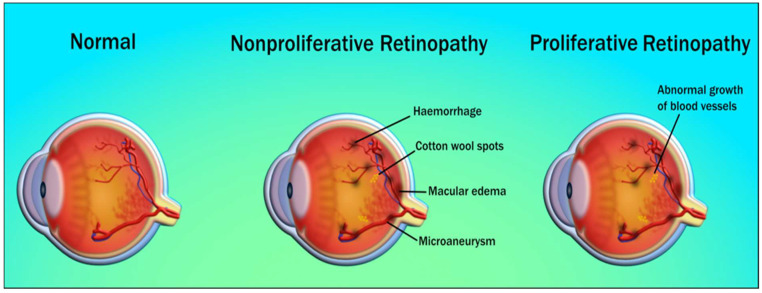
Stages of diabetic retinopathy (courtesy of AtheroPoint, Roseville, CA, USA; permission granted).

Different stages of DR throughout disease progression can be classified based on retinal imaging as mild, moderate, severe non-proliferative DR (NPDR), and proliferative DR (PDR) [78] (Figure 4). Glycation is the *microvascular* abnormality that leads to microvascular leakage, causing NPDR, and microvascular occlusion causes PDR [79]. Microaneurysm, along with blot hemorrhage, is observed in mild NPDR. Moderate NPDR is characterized by hard exudates in the retina, while severe NPDR has multiple hemorrhages and soft exudates [80]. In PDR, the new blood vessels do not supply enough nourishment to the retina. Further, they are soft, fragile, and at high risk of rupture and bleeding, causing severe vision loss or even blindness. Advanced forms of PDR may also lead to retinal detachment and blindness [81]. An important additional complication of the DR is diabetic macular edema (DME) [82], which reflects the main reason behind the loss of vision and blindness in DR patients. The macula is a part of the eye required for central vision if the leakage of fluid happens in the macula (specifically to the fovea) it causes swelling, affecting the primary area of focus [83]. DME occurs as a different severity of DR, which is both NPDR and PDR [84].

A change in the structure and cellular content of the microvasculature such as atherosclerosis is a sign of early DR [85,86]. Thus, the pathogenesis of DR has several contributing factors that are driven by atherosclerosis. Several studies have shown that endothelial permeability, neo-angiogenesis, and plaque micro-vascularization are all influenced by a blood vessel’s vasa vasorum (a network of small blood vessels that supply the walls of larger blood vessels) [87]. Recent pieces of evidence suggest that in patients with diabetes, vasa vasorum shows evolutionary changes [88]. It is the same as the beginning stage of the retina, in which endothelial dysfunction and loss of capillaries predominate [88]. This results in an unstable plaque and favors plaque rupture. As discussed earlier, hard exudates present in the moderate stages of NPDR also account for their association with CVD and plaque formation [84]. Hard exudates appear in the retina due to leakage of lipids and proteinaceous material through the endothelial barrier. It is also responsible for the appearance of plaque in large arteries [89]. Elevated blood pressure has also been associated with DR and is a significant biomarker in atherosclerotic disease [90].

According to another study [88], there is a link between diabetes and CVD, high plasma LDL cholesterol, and proteinuria. The development and progression of retinopathy may be more severe in patients with diabetes and signs of atherosclerosis, necessitating more frequent examinations and therapies in these patients. As a result, it is necessary to develop appropriate treatment choices.

### 3.1. The Biological Link between DR and CVD

**Figure 5:**
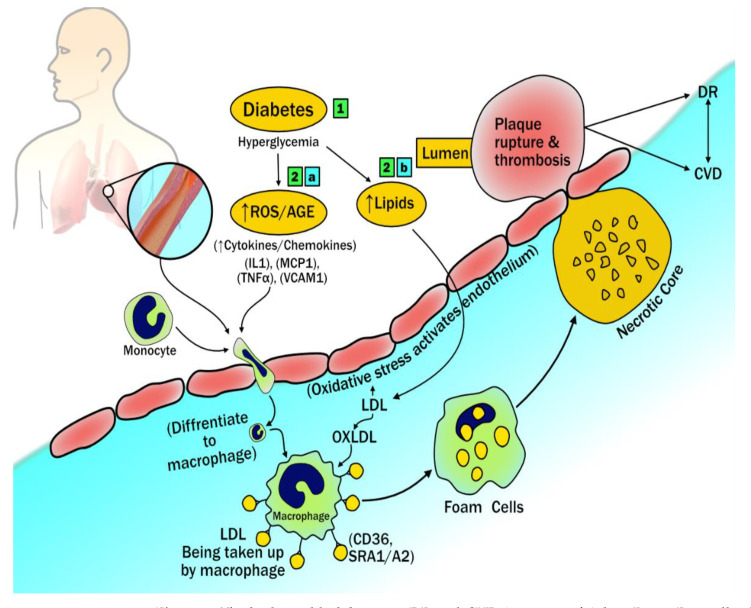
The biological link between DR and CVD (courtesy of AtheroPoint, Roseville, CA, USA; permission granted).

A vascular relationship cause exists between DR and CVD, it is observed that retinopathy is a microvascular dysfunction caused due to endothelial dysfunction that results in arteriolar wall leakage [91]. These small arteriolar and capillary bed leakage causes retinopathy and nephropathy. However, large arterial wall leakage causes lipid accumulation, consequently leading to a pathogenic cascade of atherosclerosis [92]. Hyperglycemia causes inflammation by releasing reactive oxygen species, advanced glycation end products, cytokines, and chemokines [93]. These collectively cause oxidative stress and endothelial dysfunction that facilitates the entry of monocytes and macrophages. Sequentially, endothelial dysfunction also helps low-density lipoprotein (LDL) particles penetrate the intimal wall of the vessel in a process called transcytosis [94]. Further, the LDL particles get oxidized and form OxLDL, due to the inflammatory markers process [95,96,97]. Additionally, endothelial dysfunction activates the scavenger receptors (SRc) known as SR-AI/II, SR-BI, and the cluster of differentiation 36 (CD36). These results in intracellular uptake of oxLDL by macrophages in the arterial intima and help in the formation of foam cells [98,99] (see Figure 5). Over time, the foam cells die, contributing to the production of interstitial collagen and elastin inside the foam cells resulting in the formation of the necrotic core [100]. Collectively, these overall sequential steps initiate the platelet aggregation and adhesion favors the atherosclerotic plaque formation causing micro and macrovascular complications [101].

Coronary artery disease is the most common type of heart disease and is often known as cardiovascular disease as well as coronary heart disease. It has been shown that diabetes can cause blood vessels to thicken, which can progress to CHD [102]. Thus, a person with DR should be at an elevated risk of CHD/CAD. To study this, we went through recent literature and found several interesting attributes. In a study by Barlovic et al. [103], it was observed that 416 CVD events occurred during 12,872 person-years of follow-up. Severe diabetic retinopathy (SDR) was seen to increase CVD risk, particularly for peripheral artery disease (PAD) in long-standing type 1 diabetes [104].

Hecke et al. [105] examined a cohort of 2237 type 1 diabetic patients in their study. After 7.9 years of follow-up, 64 people had died and 128 people had new CVD. People who had nonproliferative and proliferative retinopathy were more likely to die from any cause and have a higher risk of having a heart attack or suffering from stroke than people who did not have retinopathy [106]. They found that people with type 1 diabetes who have non-proliferative or proliferative retinopathy have an increased risk of all-cause death and new CVD. Another study by Pradeepa et al. [107] showed that in South Indian patients with type 2 diabetes, the prevalence of CAD was significantly higher in patients with DR compared to those without. In subjects with glycated hemoglobin (HbA1c) levels > 7% (*p* = 0.002), a significant association was observed between DR and CAD. Some people who had eye-bleeding or microaneurysms had a higher risk of having a heart attack, and those who had cotton wool spots had an increased chance that they would have a heart attack or have another stroke. The same study by Kawasaki et al. [108] concluded that type 2 diabetic patients with even a mild stage of DR, such as dot hemorrhages are already at risk of CHD. Ellis et al. [109] in their study suggested that understanding the link between DR and CVD would lead to refined treatment strategies leading to personalized treatment strategies. In another study, by Cheung et al. [110], out of 214 participants that had DR, there were 209 CHD events. The presence of DR was linked to a two-fold increase in the incidence of CHD events and a three-fold increase in fatal CHD events. Table 1 presents the link between DR and CHD.

Um et al. [111] explained that people with type 2 diabetes and PDR had a more severe coronary artery calcification and both were more likely to have CHD, compared to patients without DR. Thus, in asymptomatic patients with type 2 diabetes, PDR can be a predictor of CHD. Xu et al. [79] concluded that DR was a risk marker for CVD. Their findings indicated that DR predicts a doubled mortality of CVD in diabetes. This clearly showed that DR was strongly related to CVD. All the above studies demonstrate our hypothesis holds that DR is responsible for the worsening of CVD.

#### Diabetic Retinopathy Imaging and Cardiovascular Disease: Establishing the Hypothesis

The human eye is a vital organ that helps in the direct and non-invasive visualization of DR changes. Direct visualization of neurovasculature of the eye can be done via non-invasive imaging modalities [26]. Several studies indicate an increased risk of CVD associated with DR patients. A study reported that retinopathy signs are associated with coronary artery calcification and may be markers for atherosclerotic disease [113]. The retinal arteriolar narrowing was observed as a marker of coronary microvascular disease [35].

Alonso et al. [37] identified that Type 2 diabetic patients with DR had more atherosclerosis in their carotid arteries. Another study reported a significant association of increased carotid intima-media thickness (cIMT) with DR and peripheral vascular disease (PVD) [114]. Concomitant diabetic cardiomyopathy was indicated in those suffering from advanced DR. These people usually have or will have chronic or recurrent heart failure [115]. Thus, we hypothesize that the condition of the heart could be altered during different stages of DR. Furthermore, patients with macular edema (ME) and proliferative diabetic retinopathy (PDR) are at the highest risk of developing CVD [84]. To classify CVD risk for different stages of DR, it is necessary to generate an output of these stages in the form of retinal scans. Therefore, retinal or ocular imaging modalities have an increasingly vital role in the management of diabetes, diabetic retinopathy, and the prognostication of associated events.

Retinal imaging is a diagnostic tool primarily used in the diagnosis of retinal diseases as well as in monitoring retinal conditions with time. The three principal technologies used in retinal imaging are (i) fundus camera imaging, (ii) scanning laser ophthalmoscopy, and (iii) optical coherence tomography (OCT).

### 3.2. Fundus Camera Imaging

**Figure 6:**
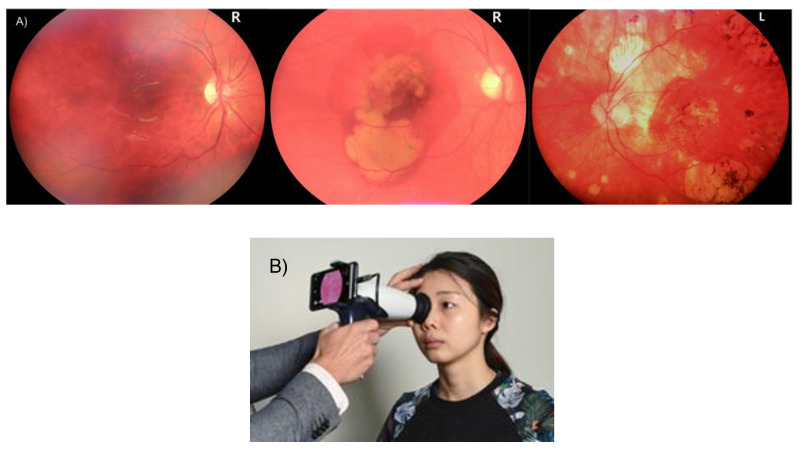
(**A**) Retinal images were taken using an IR camera [120]; (**B**) imaging using nun IR portable fundus camera [120] (Courtesy of oDocs Eye Care, Dunedin, New Zealand, reproduced with permission).

Thirty- to fifty-degree field-of-view images were provided by standard fundus photography. This includes the macula and optic nerve [116]. 3D, semi-transparent, and retinal tissues projected onto the imaging plane are acted upon by reflected light to generate two-dimensional (2D) representations [117]. Fundus camera imaging has been used extensively in DR imaging [33,118]. It provides imaging of blood vessels, lesions, hemorrhages, and exudates that are pre-symptomatic stages of retinopathy. The standard ultra-wide field, fundus autofluorescence, and smartphone-based fundus photography are several types of fundus imaging techniques [119] that are quick and simple. It covers a larger retinal field and has high patient compliance (see Figure 6). However, 3D layer visualization is not possible for some pathologies like macular edema and age-related macular degeneration, but using such 3D scans provides a quick diagnosis. Fundus imaging has advantages on the cost side, being usually a quarter of the price of an OCT scanner. Recently, cost-effective smartphone-based fundus imaging cameras have been developed that can provide good-quality retinal images.

### 3.3. Optical Coherence Tomography

**Figure 7:**
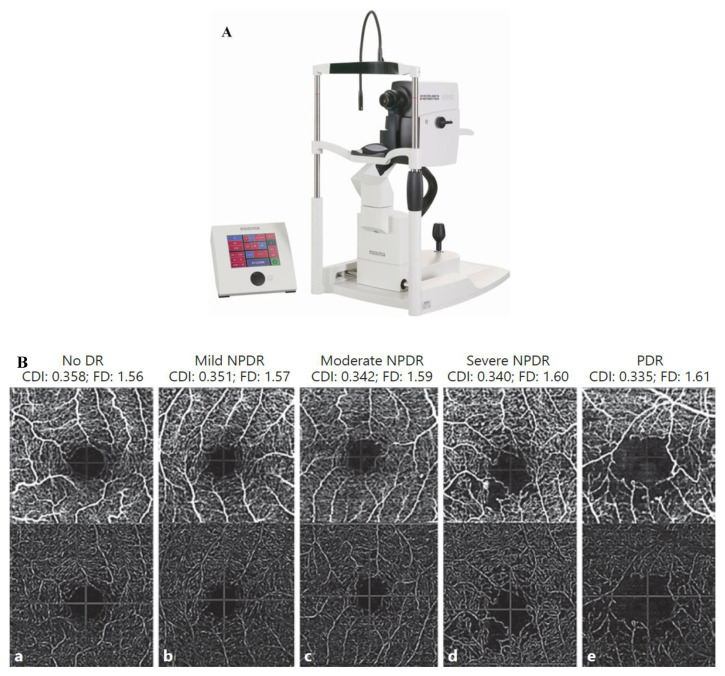
(**A**) HRA + OCT imaging with a Spectralis HRA+ device Binarized optical coherence tomography pictures with varying degrees of DR severity, as well as non-segmented angiograms, are shown in (**B**). (**a**) There is no DR. (**b**) Mild NPDR if any. (**c**) NPDR of a moderate level. (**d**) A very bad case of NPDR. (**e**) It is a PDR and it is important to note that the following CDI and FD values are the same: CDI is 0.358 and FD is 1.56, CDI is 0.351 and FD is 1.57, CDI is 0.342 and FD is 1.59, CDI is 0.340 and FD is 1.60 and CDI is 0.335 and FD is 1.61. Nonproliferative diabetes retinopathy is referred to as NPDR, while proliferative diabetic retinopathy is referred to as PDR.

OCT is a reproducible, non-invasive imaging modality that allows easy detection [121]. It has provided new areas of understanding in ophthalmology. This optical scanning technique uses near-infrared light and can be thought of as “optical ultrasound” when interpreting these scans [35]. OCT images are high-resolution scans (1–15 mm) with a penetration depth of 2 to 3 mm in human tissue [36] (see Figure 7). It gives a cross-sectional view of internal retinal structures and helps in detecting possible markers of neurodegeneration.

### 3.4. Optical Coherence Tomography and Angiography

OCT does not directly measure blood flow velocity, distinguish arteries and veins, or detect vascular permeability changes. In addition, hence ICGA and FA remain common methods to visualize blood flow [116]. However, FA and ICGA are slow and cannot produce topographic 3D images. The advent of OCTA in 2012 revolutionized ophthalmology [117]. It aids in the non-invasive examination of retinal and choroid anatomy and vasculature [26]. It helps in the non-invasive evaluation of the structure and vasculature within the retina and choroid [26]. Thus, with help of these imaging techniques, we can effectively access DR and associated CVD risk. Table 2. Shows the difference between FI and OCT.

### 3.5. DR and CVD: Does Our Hypothesis Hold True?

The advances in retinal imaging technology and its power to diagnose DR give a very strong edge for risk stratification in DR. The question, therefore, arises if *patients suffering from DR can be used for direct CVD evaluation and risk stratification, or do all the grades of DR increase the risk of CVD?*

A recent study, called Action for Health in Diabetes (AHEAD) between DR and CVD, was conducted on type 2 diabetes patients having a cohort size of 4098 participants [122]. The authors showed that there was an increase in CVD composite that affected the microvascular disease (MVD) having the following statistics [(HR 1.34, 95% CI 1.11–1.61), CAD (HR 1.24, 95% CI 1.01–1.52), stroke (HR 1.55, 95% CI 1.03–2.33), and cardiovascular mortality (HR 1.26, 95% CI 0.72–2.22)]. This was clear evidence of worsening CVD due to DR progression. There was another recent study that showed a reduction in LV function due to diabetes-related microvascular complications, which was evaluated using the global longitudinal strain (GLS) [29]. Asymptomatic patients with DM had reduced GLS and were independent of other cardiovascular risk factors. Microvascular problems were more prevalent in patients with non-obstructive coronary artery disease. Furthermore, the burden of microvascular problems was linked to a higher load of coronary artery plaque burden (CAPB).

In another study, conducted on a cohort from China having type 2 diabetes mellitus (T2DM), it was shown that ASCVD was strongly associated with DR [27]. The authors showed that the association of DR with ASCVD was significantly higher compared to patients with non-ASCVD (ChiSquare: χ^2^ = 5.805, *p*-value = 0.016). The authors further demonstrated that DR was an independent statistical indicator in the presence of ASCVD having the odds ratio (OR) (95% CI): 2.321 (1.152–4.678), *p*-value = 0.018.

Further, only PDR was linked with ASCVD [OR (95% CI): 8.333 (1.813–38.304), *p*-value = 0.006]. The connection remained after adjusting for ASCVD risk variables [OR (95% CI): 7.466 (1.355–41.137), *p* = 0.021]. Future risk of CVD, MI, and CHF [30], was associated to DR severity in 2020. DR enhanced the incidence of CVD, MI, and CHF mortality. In their study, there were 77,376 patients, including 59.8% men, 31.28% non-Hispanic Whites, and 41.48% Hispanics. Minimal NPDR increased the likelihood of CVA (1.31; 95% CI, 1.18–1.46), MI (1.30; 95% CI, 1.15–1.46), and death (1.29; 95 percent CI, 1.19–1.40). HR 1.15; 95% CI 1.05–1.25. Patients with symptomatic NPDR and proliferative diabetic retinopathy showed increased mortality (HR, 1.55; 95% CI, 1.32–1.82); (HR, 1.92; 95 percent CI, 1.57–2.34); CHF: HR, 1.96; 95% CI, 1.47–2.59; and death: HR, 1.87; 95% CI, 1.36–2.56.

### 3.6. Descriptive Analysis Validating the DR-CVD Hypothesis

Mimoun et al. [123] showed the relationship between the retinal microvasculature challenges and (a) white matter lesions in the brain leading to stroke and (b) coronary calcification leading to heart failure. These retinal microvasculature challenges consisted of reduced (i) arteriolar diameter, (ii) venular dilatation, and (iii) retinopathy lesions. The authors showed ventricular dilation was due to the presence of diabetes, obesity, and metabolic disorders. The authors further presented that retinopathy is correlated with cerebral white matter lesions in the brain leading to stroke. This was evidenced by an MRI of the brain. On the heart side, the authors showed that these microvasculature challenges were related to coronary calcification leading to heart failure. From this study, we conclude that there is a direct relationship between retinal damage due to diabetes and coronary artery calcification. Thus, since carotid artery disease is a surrogate marker of coronary artery disease, we can adopt carotid artery disease biomarkers for CVD risk assessment in diabetic retinopathy patients.

Flammer et al. [124] in their study concluded that there are various common characteristics observed between the eye and the heart. The authors showed that an increase in retinal vein occlusions or retinal arterial, cataracts, age-related macular degeneration, and an increase in intraocular pressure (IOP) trigger atherosclerosis and risk factors like dyslipidemia, diabetes, and systemic hypertension. This directly leads to cardiovascular diseases. Another study by Seidelman et al. [125] found a link between bigger retinal venules and narrower retinal arterioles and cardiovascular health. Pooled Cohort Equations (PCE) were used by the authors to look at the risk of CVD. This study took 10,470 men and women from the Atherosclerosis Risk in Communities (ARIC) study who had never had heart failure or heart disease before. People who took part in this study were tracked for 16 years. They had 1779 heart disease events, 548 ischemic strokes, 1395 heart failure events, and 2793 deaths. The authors proved that the hypothesis was correct by comparing the hazard ratio (HR), 1.13; the 95 percent confidence interval (CI), 1.08–1.18; the HR, 1.18; the CI, 1.07–1.31; the HR, 1.10; and the CI, 1.00–1.20 per 1-SD increase and narrower retinal arterioles and venules (HR, 1.06; 95 percent CI, 1.01–1.11; HR, 1.14; 95 percent CI, 1.03–1.26; and HR, 1.13; 95 percent CI, 1.03–1.24 per 1-SD decrease). We think that eye diseases, like narrower retinal arterioles and wider retinal venules, raise the risk of ischemic stroke and coronary artery disease.

It has been observed that CHF impairs retinal microvascular dilatation in response to flicker light. It was shown that retinal vessel analysis can be a new and valuable tool to non-invasively assess microvascular problems in heart failure. In their investigation, Freitas et al. [126] looked at the hemodynamics of the ocular artery in individuals with congestive heart failure. Comparing patients with chronic heart failure with Liew et al. [92] explain the connection of retinal vascular fractal dimension (D_f_) with coronary heart disease mortality offering a global network of vascular architecture. They looked at D_f_ and 14-year CHD mortality in a population-based study of 3303 people aged 49 or older. The development of the clinical cardiovascular disease may be a result of suboptimal microvascular branching. Naegele et al. [127] the control group, this study found that the occurrence of orbital vasoconstriction in response to reduced cardiac output was observed in the former group. The investigators also discovered that a lower diastolic velocity and a greater resistance index in the ocular artery were associated with coronary heart disease. As a result, the authors concluded that further research into the impact of these discoveries on the anatomy and function of the optic nerve head is warranted. Liao et al. [128] concluded in their findings that generalized narrowing of the retinal arterioles independent of blood pressure and other vascular factors is related to greater stiffness of the carotid arteries. This further supported the relation between macrovascular and microvascular disease in stroke pathogenesis.

McClintic et al. [129] presented a literature survey to study the relationship between abnormalities of retinal microvascular and coronary heart disease. Since the traditional CVD risk, stratification had limitations; the authors used retinal vasculature as a screening mechanism for CVD risk stratification. The authors particularly proposed a change in the guideline for women who had a low risk for CVD, which is typically ineffective by traditional risk methods. Table 3 provides a summary of the above-listed findings.

## 4. Carotid Imaging for CVD Risk Assessment in DR Patients

**Figure 8:**
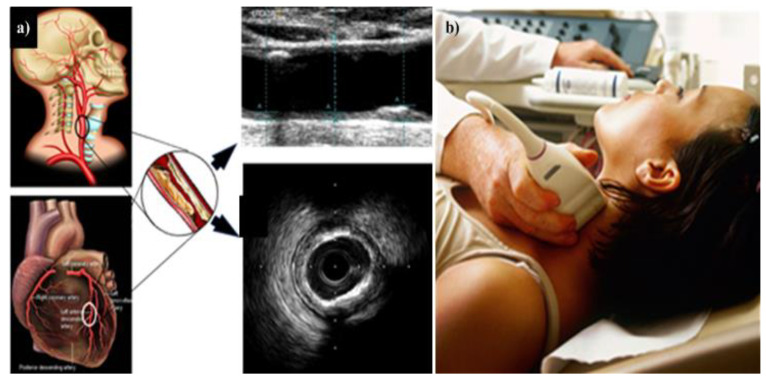
(**a**) The carotid artery is employed as a proxy for coronary artery disease. (**b**) Imaging gadget with a linear ultrasound probe scanning the carotid artery. (Courtesy of AtheroPoint, Roseville, CA, USA; produced with permission).

DR can act as an indicator not just for cardiovascular or coronary artery diseases, but also for cerebrovascular diseases. Figure 8 shows how the link between carotid ultrasound and coronary artery disease, both having the common thread of atherosclerosis. Figure 8a shows the visualization of the carotid and coronary scans using radiation-free ultrasound scans. Figure 8b shows the typical low-cost and portable ultrasound measurement device used for screening the carotid arteries.

Thus, there are two reasons for studying the surrogate markers for CVD. First, it has already been proven that DR is associated with carotid artery disease. Second, DR is associated with coronary artery disease which in turn is associated with a carotid atherosclerotic disease that acts as a surrogate marker for coronary artery disease [131,132].

In support of the former case, several studies have been published that links DR to carotid artery disease [27,30]. Similarly, several important studies have been published that link carotid artery disease to coronary artery disease [131,132,133]. Thus, this section is focused on (a) the relationship between DR and carotid artery disease and (b) carotid artery as a surrogate marker for coronary artery disease.

### 4.1. DR and Cerebrovascular/Carotid Artery Disease

A recent study investigated whether minute retinal microvascular changes indicate DR, and if this could cause arterial stiffening [28]. The authors hypothesized that retinal microvascular dysfunction may be seen in patients with carotid stiffness. Their findings indicated that stiffness was associated with a decreased ability of the retina to dilate in response to flickering light. Additionally, this link was shown to be greater in persons having type 2 diabetes. A further study by Lee et al. [134] explained the disruption of the common carotid artery (CCA) could lead to retinal ischemia. This is because of the ophthalmic artery (OpA), which is a retinal artery supplying blood from the internal carotid artery (ICA). The retinal blood supplying vessel is the OpA.

Several studies by Drinkwater et al. showed that carotid artery disease is independently associated with the retinal microvascular disease as assessed by OCTA in type 2 diabetes [135,136,137]. In another study, by Lu et al. [138], a link between time in range (TIR) and macrovascular disease was suggested after seeing an increase in cIMT associated with TIR. Meanwhile, DR was seen to be an independent predictor of subclinical cardiovascular disease [38]. With the risk of carotid artery disease, it is also vital to look at coronary artery disease. Since both these diseases hold a link, this will cause the condition of DR to worsen.

### 4.2. Carotid Artery Disease—A Surrogate of Coronary Artery Disease or Cardiovascular Disease

**Figure 9:**
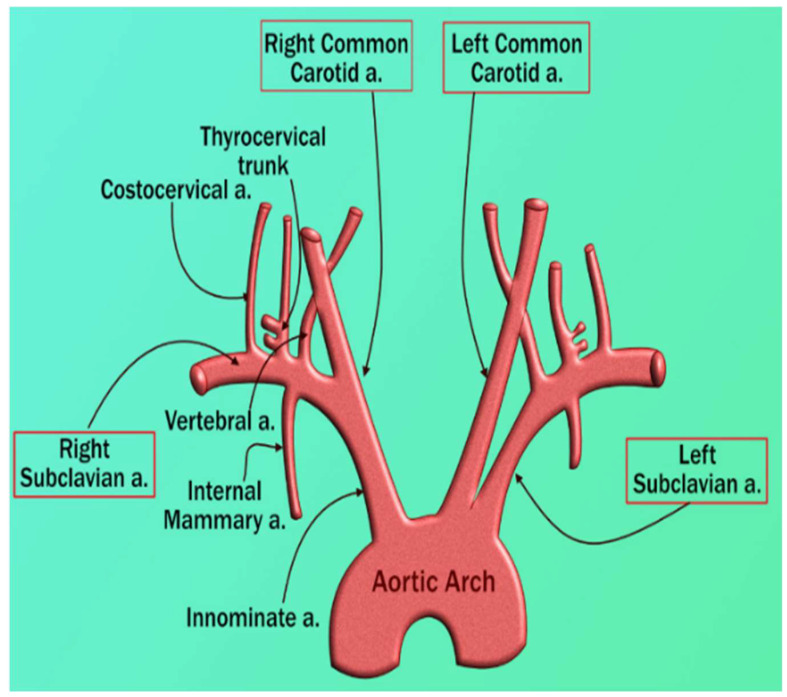
The origination of the left and right carotid arteries (courtesy of AtheroPoint, Roseville, CA, USA; reproduced with permission).

There is a risk to the heart and brain due to vascular diseases [139,140]. Both carotid and coronary arteries have a common genetic makeup. This link is well established due to the similarity of the structure between the aortic arch, coronary artery, and carotid artery. Even though these arteries stem from a different major artery, they follow symmetrical courses (see Figure 9). Thus, carotid artery disease could be considered a surrogate biomarker for CAD [141,142].

MRI [143,144,145], CTA [146], OCT [147], PET [148,149] are some of the common imaging modalities utilized for carotid artery imaging and angiography carotid screening. All these modalities have the ability to image carotid plaque [3,149]. Ultrasound is the most popular, easy-to-use, high-resolution, economical, and user-friendly image acquisition modality able to identify plaque [150,151,152]. As a result, it has broad application for routine preventative screening of atherosclerotic plaque and risk assessment for cardiovascular disease [54,151,152,153,154,155]. It is possible to use automated systems to figure out the imaging phenotype [156,157,158,159]. They can be checked out even more with CT, MRI, or the gold standard [150,160]. Carotid ultrasound images from the far wall, as well as their structure [161], can show cIMT, the total area of plaque in the carotid artery, the carotid artery intima-media thickness variability (IMTV), and the morphology and height of the plaque. They play a big role in the prediction of CVD [54,162].

Combining these carotid image-based phenotypes with traditional cardiovascular risk variables [55,150,152,163] has been shown to improve CVD risk prediction. According to several studies, cIMT and carotid plaque progress annually [164,165,166,167,168,169]. Blood biomarkers and carotid ultrasonography have been used to predict 10-year risk and also improve plaque detection and atherosclerotic disease monitoring [170]. Various studies explain the link between carotid and coronary artery disease with help of AI it is possible to predict coronary artery disease by using carotid artery [171,172,173,174].

## 5. Artificial Intelligence and Its Role in Cardiovascular Disease Risk Stratification

Several of the existing and most recent guidelines by the American College of Cardiology (ACC) and American Heart Association (AHA) recommend the use of some algorithms to perform CVD risk assessment. The estimated risk using risk calculators is used to initiate statin therapy in patients to control their overall risk of CVD (Table 4).

Several CVD risk calculators have been developed based on this concept, all within a statistical framework.

The Framingham risk score [181], the systematic coronary risk evaluation score (SCORE) [182], QRISK3 [183], and the pooled cohort risk equation created by the American College of Cardiology and the American Heart Association [175] are the most frequently used CVD risk calculators.

Because they were developed based on specific ethnic cohorts, when applied to diverse ethnic populations, they can either underestimate or inflate the risk of cardiovascular disease. These calculators were built using regression-based techniques that can handle a limited set of risk predictors.

- Because these CVD risk calculators were developed through the use of regression-based approaches, they assume that there is a linear relationship between the risk predictors and the endpoints. Because of this constraint, a complicated non-linear association between the risk predictors and the endpoints is not taken into consideration.
- The final and most significant difficulty is that such conventional risk factors are exclusively reliant on traditional risk variables, which do not provide any information on atherosclerotic plaque burden in the first place. It is possible to overcome this difficulty by utilizing low-cost imaging methods.

**Figure 10:**
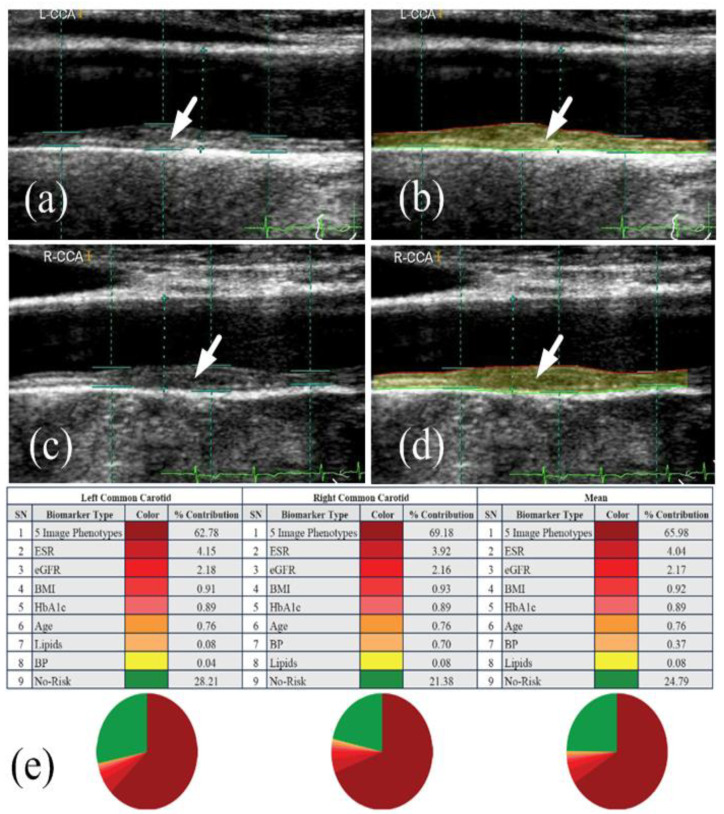
Risk predictors make up a big part of a person’s 10-year CVD risk profile when they’re looked at for the left common carotid artery (**a**,**b**), right common carotid artery (**c**,**d**), and the average of left and right common carotid artery (AtheroEdge 2.0) (**e**). This figure was made with permission [201] by AtheroPoint, USA. (Courtesy of AtheroPoint, Roseville, CA, USA; reproduced with permission).

Due to these issues, a more reliable and precise CVD risk prediction model is required. Traditional risk calculators can be improved by including image-based phenotypes in CVD risk prediction models. Suri et al. [153,184,185,186,187] have attempted this by combining automated carotid ultrasonography image-based phenotypes with traditional risk variables to produce integrated CVD risk. Ten-year CVD risk was calculated utilizing carotid ultrasound image-based phenotypes and traditional risk variables (Figure 10). The pie figure shows the independent contribution of conventional risk variables and carotid imaging phenotypes to 10-year CVD risk. To improve overall accuracy and address other issues, improved risk prediction algorithms are required.

**Figure 11:**
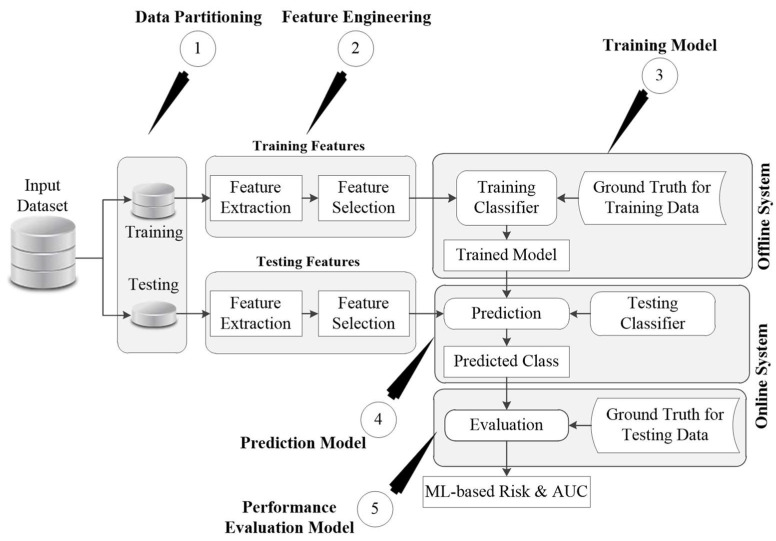
The generalized architecture of the ML-based system.

AI-based algorithms have proved themselves to be superior to the existing CVD risk calculators [188,189,190]. This is the reason for the growing interest of clinicians in exploring the potential of AI in dealing with several healthcare problems, including the CVD risk assessment. AI is primarily categorized into two types of algorithms: machine learning and deep learning algorithms. Both of these algorithms require large datasets under a big data framework to build their internal models and provide accurate risk assessment [191]. Machine learning algorithms require a series of pre-processing steps that involve data cleaning, noise reduction [192,193], feature extraction, and feature selection. Several good examples can be seen for different disease characterization such as diabetes [194], lung [195], thyroid [196,197], liver [198,199], breast [200], and coronary [195]. Figure 11 shows the general framework of any ML-based algorithm.

The generalized architecture is commonly divided into two parts that are comprised of an offline model and an online model. The offline model deals with the training of an ML-based algorithm using the risk factors and endpoints and generates the offline coefficients. This will then be used under the online model to transform the unseen risk predictors into final CVD risk labels. Both of these offline and online models require handcrafted features for the training and prediction of labels. In CVD risk assessment, such features can be derived from the patients’ demographic and clinical parameters, including laboratory-based blood tests, electronic health records, and imaging modalities. Compared to the existing CVD risk assessment, calculators such as Framingham risk score (FRS), Pooled cohort risk equation (PCRE), and QRISK3 calculators that can handle only a limited set of risk factors, ML-based algorithms can deal with a much larger number of risk predictors at the same time. Figure 11 shows the generalized architecture of the ML-based system.

ML-based algorithms make the final prediction based on several linear and non-linear patterns available within the input risk predictors. This is a key specialty of AI-driven algorithms, which makes them distinct from several conventional CVD risk calculators. Commonly used and popular ML-based algorithms are the support vector machine, random forest, decision tree, and extreme gradient boosting [202].

**Figure 12:**
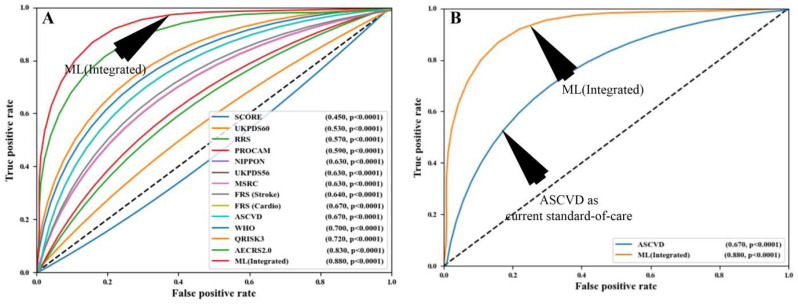
Comparing the ML-based CVD risk assessment using AtheroEdge™ 3.0_ML_ with (**A**) 13 types of CCVRC and (**B**) the standard-of-care ASCVD calculator (produced with permission [207]).

Nearly all ML-based algorithms can efficiently distinguish between the low-risk CVD patients and the high-CVD-risk patients [203]. In terms of multiclass endpoints, the ML-based algorithms have provided better risk stratification compared to the conventional CVD risk calculators [185]. Besides this, the ML-based algorithms also efficiently differentiate symptomatic and asymptomatic carotid atherosclerotic plaques [204,205]. Recently attempts were made to combine the traditional CVD risk calculators with the carotid atherosclerotic plaque-based phenotypes [206]. This combination is referred to as integrated risk predictors. Such integrated risk predictors have shown high CVD risk prediction ability under the ML framework compared to using the traditional risk factors alone [154]. When compared against the 13 existing CVD risk calculators, such integrated feature-based ML systems reported superior performance [207]. This can be seen in Figure 12, which shows the comparison between the ML algorithms and statistical calculators.

It was found that AI-based algorithms had a better overall risk-strategy accuracy of 92.52 percent than the 13 types of CCVRC. This was more than any of the 13 types. Other people have shown that machine learning can be used to make better risk predictions. They used carotid ultrasound plaque attributes to enhance the risk prediction precision [202,208]. One more ML-based study, by Kakadiaris et al. [190] and Weng et al. [188], also found that ML-based algorithms were better than conventional CVD risk calculators based on statistics.

Machine learning has proved to be a boon not only in CVD risk stratification, but also in several other areas, including benign and malignant tumor identification [209], characterization of intra-nodular vascularization of thyroid lesions [210], psoriasis identification [211], and so on.

**Figure 13:**
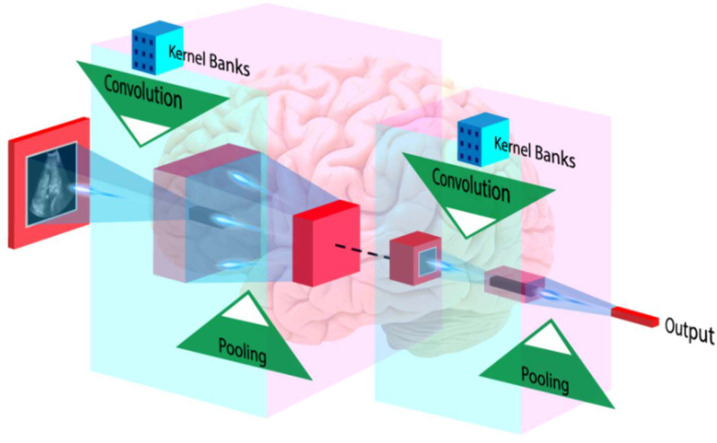
A general architecture of CNN used in medical image analysis application (courtesy of AtheroPoint, Roseville, CA, USA).

In addition to ML, DL-based algorithms are also powerful in making an accurate and reliable diagnosis. DL techniques are the extension of a classical artificial neural network and can efficiently be used for medical image analysis, including feature extraction and classification [212]. Unlike ML-based algorithms, DL algorithms extract features by themselves and perform classification or prediction tasks [213]. In medical imaging, a popular algorithm called the convolutional neural network (CNN) has been getting a lot of attention. This algorithm is based on Deep Learning. CNN can find more high-level features than artisanal ones, and it can use them to make medical diagnoses [45,214]. In Figure 13, the input image is convolved using a set of kernels (also called filters) that extract multiple high-level patterns from the image.

A pooling operation selects the meaningful and dominant features. During CNN training, the backpropagation algorithm learns the overall coefficients of all kernels. Lekadir et al. [215] recently employed CNN to classify carotid ultrasound pictures into lipid, fibrous, and calcified plaque. CNNs have also been used to assess carotid phenotypes such as intima-media thickness and lumen diameter [216,217,218].

Recently, Rim et al. [47] used the DL-based algorithm to predict the CAC score from retinal images. The authors proved that the CAC score measured using retinal images was comparable to the CT-derived CAC scores. Cheung et al. [48] used the DL-based algorithm to assess the CVD risk via the measurement of retinal vessel caliber. Besides this, DL-based algorithms have been widely adopted for the screening of DR patients [51,52,219]. Thus, it is very evident that both AI-based algorithms could be used for accurate CVD risk assessment as well as for automatic DR detection. Furthermore, along with traditional risk factors, the integration of carotid ultrasound plaque phenotypes could be used for preventive screening of patients in atherosclerosis and risk estimation.

## 6. DR/CVD in the COVID-19 Framework

COVID-19 has caused massive global death [219]. It has caused over 5.7 million fatalities globally [220]. The coronavirus disease that originated in 2019 is named COVID-19. It is triggered by SARS-CoV-2 [221] and damages numerous routes [222]. Diabetes type 1 and 2 patients had an increased incidence of COVID-19 [223,224]. Individuals with existing CVD are also at risk of COVID-19-related problems [225,226,227,228,229]. Several investigations have linked (SARS-CoV-2) and ocular symptoms [227,230,231]. COVID-19 symptoms range from mild to severe; one of these symptoms includes anosmia (loss of smell). Various studies suggest that anosmia is due to the effect of the virus on the olfactory bulbs [230]. It has been very well established that olfactory impairment is associated with diabetes and related microvascular complications [231]. In COVID-19, patients with DR have an elevated risk of unfavorable conditions [232]. Costa et al. [233] recently examined individuals who had recovered from the COVID-19 acute phase, 15.6% of whom had DR.

With the ongoing pandemic, it is critical to evaluate these patients’ situations. Several studies have reported the difficulties that several countries face in doing DR screening amid the pandemic [234,235]. Telemedicine was reported to be a useful solution for DR [236]. Telemedicine platforms specific to ophthalmology practice have also been developed [158,237,238]. It is, therefore, vital to opt for a low-cost, preventive screening tool to provide effective treatment strategies and risk stratification for these patients as soon as possible. These patients are risking life and limb in the ongoing pandemic [201,224].

### 6.1. Adverse Effects of COVID-19 on DR Patients

This section shows how DR was affected during the COVID-19 period. The importance of DR during pandemic times has risen several folds. DR management has been suspended because of SARS-CoV-2, which has hampered access to diabetes-related medical consultations and retinal tests [237]. Many DR screening services and referral initiatives have been negatively impacted by the COVID-19 pandemic [238].

In the COVID-19 period, diabetes was related to a greater probability of unfavorable DR outcomes due to the worsening of preexisting pulmonary microcirculatory abnormalities [239].

Ahmed et al. [240] noted that the COVID-19 pandemic has altered real-world practice patterns in DR management. Due to a change in these patterns, intravitreal injections for the treatment and management of DR were reduced significantly. Similar findings were seen in a study conducted by Chatziralli et al. [241], which concluded that there was a significant postponement in patient care that further led to worsening of visual acuity outcomes in patients with DR. Thus, these studies clearly show the health hazard for DR patients during COVID-19 period. Das et al. [242] found a rise in patients with proliferative DR (56%) and sight-threatening DR (79%) as well as vitreoretinal operations (31%), and intravitreal injections (19%). During the lockdown, the number of blind patients grew. This highlights the need for proper DR treatment during COVID-19. Dwairi et al. [243] found that delaying or interrupting a key operation for DR patients could harm their visual prognosis. The authors also advised residential monitoring, “treat and extend” strategy, portable OCT testing, and more long-acting anti-VEGF (vascular endothelial growth factor) medicines.

COVID-19 individuals had an intraocular infection, according to Nayak et al. [244]. This infection was mostly fungal; the authors concluded that routine eye exams were required during COVID-19. Due to the relevance of DR during pandemics, mobile applications were developed. Khurana et al. [245] compared the use of a mobile application called Checkup Vision Assessment System to routine VA reference tests in the clinic. The authors demonstrated the need for such a smartphone device, especially during the global pandemic. Saxena et al. [246] highlighted the presence of vitamin D supplementation by evaluating serum vitamin D levels during the COVID-19 period. When Vitamin D levels are below 10 ng/mL, the study found a relationship between diabetes and COVID-19. Several research has come out with new treatments to overcome resource and geographic constraints. Walsh et al. [247] stated that investment in national strategic alliances and technology can assist in promoting health and ophthalmic care. Teleophthalmology will thus be vital in the development of eye care. SFI is a technique developed by Kumari et al. [248] that allows patients to take photos of their retinas, addressing the barriers to availability and affordability in an era of the pandemic.

Thus, we conclude that DR and COVID-19 run side by side and cannot be ignored. Therefore, we need the CVD estimation of patients who are DR affected during COVID-19. Further, it should be noted that efforts toward treatment and manifestations on DR patients were very active during the COVID-19 period. This demonstrates the importance of DR during the COVID-19 pandemic, which is still active. Our group has been very closely monitoring the comorbidities during the long COVID-19 period [249]. This further asserts our assumption that DR cannot be ignored, and we must study the relationship between DR-CVD during the COVID-19 period. In the next section, we present the link between DR and CVD during COVID-19 times. From the above discussions, we finally conclude that during the COVID-19 period, diabetes and DR were both severely affected. This has a direct bearing on the heart and CVD.

### 6.2. Relationship of DR and CVD during the COVID-19 Period

**Figure 14:**
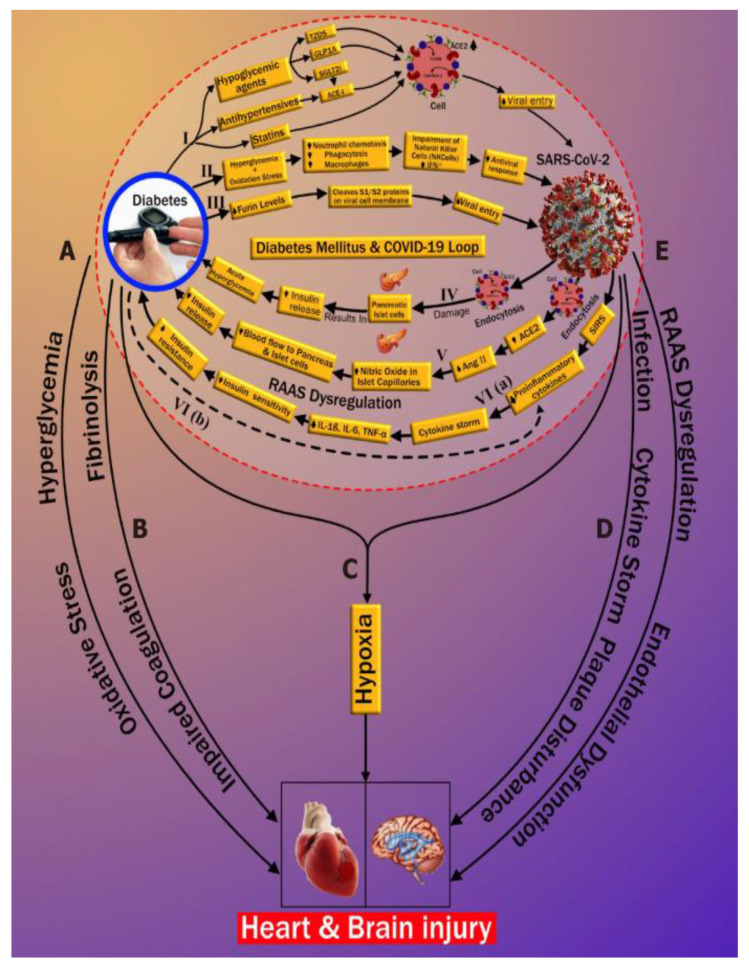
The diabetes–coronavirus disease relationship with Heart and Brain.

Diabetes has a strong relationship with COVID-19. Very recently, our group published an international journal showing the relationship between Diabetes and COVID-19, which inspired us to work on Diabetes-DR-CVD and COVID-19 [9]. This is the first journal paper that links the “bidirectional nature of diabetes” with COVID-19, called the process of “diabetes ketoacidosis (DKA)”. We show here how COVID-19 increases the HbA1c and how the viral entry of COVID-19 increases in the presence of diabetes [250]. This can be seen in Figure 14 below, showing DKA.

Patients with COVID-19 who had no previous history of diabetes developed significant consequences such as DKA. DKA arises due to the overproduction of opposing regulators, which favors ketones [251]. Low insulin levels also cause it. DKA is more common in type 1 DM patients [40]. It can also happen in people with type 2 diabetes [252]. The three paths of new-onset DM or progression of pre-existing DM after COVID-19 infection are shown in Figure 14 (pathways IV, V, and VI). As shown in Figure 14, the diabetes–CVD link leads to CVD and stroke [173]. This is the most important figure in the DR-CVD investigation. This shows the importance of CVD work during COVID-19 times and the link between uncontrolled diabetes, CVD, and COVID-19. COVID-19 and CVD have another significant component [41]. Thus, it is very important to link COVID-19 with DR and CVD. COVID-19 has serious impacts, especially on patients with underlying conditions like CVD, diabetes mellitus, and hypertension. In individuals with the underlying disease, cardiac events worsen [253]. Many hospitalized patients reported heart damage in the range of 12–26%. The infection’s cytokines may alter the patients’ intramural coronary arteries. Cardiovascular disorders affect ARDS in COVID-19 afflicted patients [254].

SARS-CoV-2 can cause complex severe ruptured plaque [255]. The enhanced instability of coronary and carotid plaques may also enhance the risk of cerebral ischemic strokes and myocardial infarctions in SARS-CoV-2 positive asymptomatic individuals [9,256]. The above discussion is a clear pathway between COVID-19 and CVD. Further note that the process of DR accelerates the worsening of CVD in the COVID-19 framework.

### 6.3. The Overall Architecture of the DR-CVD System in the COVID-19 Framework

**Figure 15:**
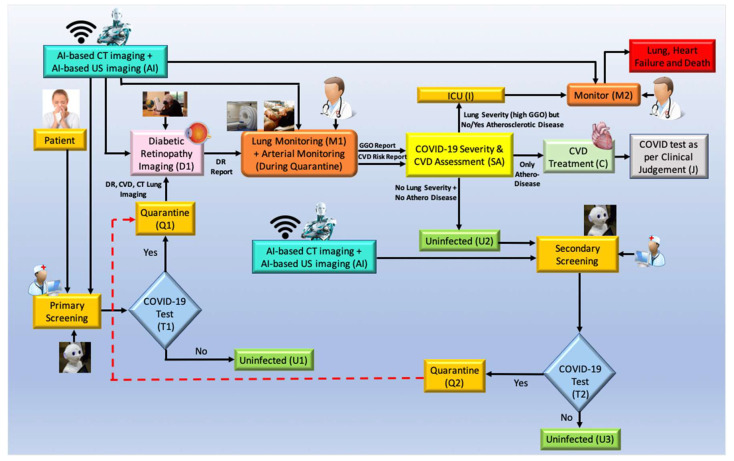
COVID-19 risk assessment for DR and CVD.

COVID-19 screening has two important components: primary and secondary. Figure 15 depicts the workflow for COVID-19 diagnosis and screening, as well as the use of AI in CVD screening. Screening: in the COVID-19 framework, a robot and AI screen the patient. The robot asks questions about basic symptoms and then determines whether or not screening tests like RT-PCR are necessary (shown as T1, diamond box). Cross-questioning begins when the patient enters the clinic or hospital. The AI-based machine intelligence system and telemedicine (TM) are used to perform the initial analysis (shown in the yellow ellipse, where the nurse and robot signs are depicted). The patient should be isolated depending on the outcomes of the T1 junction RT-PCR test (diamond box). The test can confirm if the patient has COVID-19 (Q1, marked yellow). The test is negative if the patient has no COVID-19 (marked green, U1). If positive, the patient can undergo Diabetic Retinopathy Imaging (D1), and based on the results obtained, it is vital to go for a CVD risk assessment.

*The outcome of the risk assessment box (SA):* During monitoring, an AI-based system assesses the patient’s risk (shown using M1). During monitoring, the doctor talks to the patient about his lung and atherosclerotic arterial health. The AI uses X-ray/CT imaging to assess lung status. Carotid ultrasonography evaluates atherosclerotic arterial status for CVD risk. (I) deteriorating lung condition and the need for ICU; (II) worsening CVD condition and requirement for CVD medications; (III) untouched.

The risk evaluation box has three outcomes (SA). (I) Ground Glass Opacities (GGO) in the patient (from the output of the monitor). When there are no atherosclerotic lesions in the carotid arteries, the person is considered severely symptomatic and is assessed in the ICU with ventilation. The lungs are slowly filling up with fluid, therefore this is a must (see Figure 15). In the ICU, the patient is constantly observed (shown by monitoring function M2). To treat CVD (II) if the patient’s lungs are stable (extremely low GGO) and the carotid arteries are plaque-burdened (C). In outcome (III), the individual has neither lung nor arterial damage, making him uninfected (U2). Since the individual was quarantined (Q1) and the risk assessment (A) was done using AI-based imaging, the doctor must assess the patient based on clinical judgment (C) (marked as J). Finally, after the individual is uninfected (U2), the extensive COVID process begins (secondary screening, marked SS). If positive, the quarantine feedback loop starts again (Q1). They are COVID-19-free if negative (U3, marked green).

### 6.4. Role of AI in CVD Risk Assessment for COVID-19 Screening

Using a machine learning technique, imaging data can be used to predict CVD risk at the vascular scale [208,257,258,259]. The goal is to obtain OCT scans to see if the person has Diabetic Retinopathy (DR). Further, if positive, the patient is at risk for CVD and should be monitored for lung and arterial function. Plaque image-based characteristics can be obtained from patient carotid scans and risk estimated using machine learning. The AI component simply requires the green trained model labeled Imaging-based AI with a robot AI logo on it. The machine learning algorithm tells the patient’s clinician (M1) if the relative risk is low, medium, or high. The patient’s CVD risk is assessed using this color-coded system (marked as SA). This can include evaluating the CT lungs for ground-glass opacity [260] or pulmonary embolism [261]. Thus, the proposal depicts AI-based CVD monitoring for pandemic diagnosis.

## 7. Critical Discussion

This review provides insight into the link between DR and CVD. The impact of DR on vision is well understood. Further, after a thorough analysis of various studies, it is evident that DR patients are at increased risk for CVD conditions. Therefore, along with DR screening, it is optimal to adopt a low-cost system to prevent the worsening of the CVD conditions of a patient. With the help of AtheroEdge™ 2.0 and 3.0, effective monitoring can be done for these patients and long-term complications can be prevented with the help of AI-based interventions in the model. Deep learning and Machine learning helps in calculating CVD risk with greater sensitivity and specificity. The model can be trained in such a way that it does not need any human intervention and results can be measured quickly. This proves to be a breakthrough in the current healthcare systems. With the help of this information, clinicians can counsel patients with vision-threatening DR and further guide them about associated CVD risk.

### 7.1. Benchmarking

After a perusal of various studies, several articles discussed the link between DR and CVD (Table 5). However, there was no such article found which was addressing all the components in our review. Alonso et al. [37] showed that DR in T2D patients with normal renal function and without CVD risk was associated with a higher plaque burden (≥2 carotid plaques) in carotid arteries. The authors conducted a cross-sectional study on 312 patients (51% men), with 153 patients with DR. The authors demonstrated the percentage of carotid plaques in DR was higher than without DR (68% vs. 52%, *p* = 0.0045). The authors used multivariate logistic regression that DR was independently associated with cIMT (*p* = 0.0176). In another study, Simó et al. [38] made an interesting remark relating to DR and CVD suggesting that, while staging diabetes, DR and CVD should never be considered as separate entities. The authors further suggested DR to be equivalent to hypertension and dyslipidemia in terms of deteriorating CVD. The authors suggested that if diabetes is not considered when evaluating CVD, the results could be biased. Finally, they concluded that all DR patients must undergo CVD screening for better risk stratification. Ting et al. [26] discussed in their chapter that retinal venules which are wider and have narrower arterioles were associated with an increased risk of CVD. The authors discussed the role of narrower retinal arteriolar caliber were significantly associated with low eGFR in CKD patients.

The authors used deep learning on fundus images and correlated them with 5-year CVD risk. Further, Gupta et al. [39] presented in their review the role of examining and working on the heart and predicting the macrovascular changes based on microvascular features and functions derived from retinal imaging. The authors showed a link between retinal imaging and the diagnosis of CVD. Since the usage of retinal scanning helps in the diagnosis of DR, age-related macular degeneration, and glaucoma, the authors linked this with heart conditions. Son et al. [22] showed the relationship between DR and subclinical atherosclerosis by using 142 subjects with T2D who are free from CVD.

The methodology consisted of (a) cIMT measurement using carotid ultrasound and (b) CVD risk using UK Prospective Diabetic Study (UKPDS) calculator. The authors observed that patients with subclinical atherosclerosis had a higher rate of hypertension and DR. Further, CVD risk was higher in subjects with subclinical atherosclerosis. The study concluded that DR is an independent risk biomarker for subclinical atherosclerosis in T2D. Hence, to our understanding, no other article has provided such valuable insights about different approaches towards the diseases that are necessary to study DR and CVD as well as provide necessary interventions in their treatment strategies.

Our previous study, Integration of cardiovascular risk assessment with COVID-19 using artificial intelligence [262], the so-called “RCM study”, also used a surrogate marker for CVD risk assessment; however, the differences between RCM study and the current study, the so-called DR study, should be noted. While the block of surrogate biomarkers remains the same in both scenarios, the fundamental difference is the key applications covered in the RCM study vs. the current study, i.e., the COVID-19 cohort vs. the Diabetic Retinopathy cohort. The patient selection criteria are based on the COVID-19 symptoms vs. diabetes symptoms. The second fundamental difference lies in the biological drive for the diseases, about acute respiratory distress syndrome (ARDS) and cytokine storm in the COVID-19 paradigm vs. atherosclerosis formation in the arterial vessels, leading to damage of coronary arteries. Another secondary difference between the two approaches is the usage of the imaging modality for image data acquisition, such as MRI/CT in RCM study vs. intravascular ultrasound (IVUS) for coronary artery imaging in DR study. While the imaging is conducted differently, the measurement criteria also subsequently change by computing 3D straining imaging for the left ventricle (LV) chamber in RCM study vs. quantification of the plaque burden in either IVUS study or carotid arterial plaque measurements or plaque burden in carotid arteries. Please note that the surrogate biomarkers for both studies utilize the B-model carotid ultrasound for plaque burden measurements, which are surrogate biomarkers for CVD/Stroke imaging. Finally, since the application is different, the workflow changes entirely between the two applications. In the RCM study, we followed CVD/Stroke risk assessment during COVID-19 times using CT imaging for monitoring the lung followed by carotid scanning for plaque build-up measurements, the surrogate marker for CVD/Stroke risk, while in the DR study, the workflow consisted of the following steps: (i) DR imaging, (ii) Lung CT imaging, and carotid arterial imaging. Thus, the COVID-19 severity report and CVD risk assessment reports were generated, followed by the manifestations.

### 7.2. Recommendations

It is clear from our review that DR patients must opt for CVD risk assessment techniques, especially the patients suffering from high risk, vision-threatening DR. Carotid imaging could be a boon for patients undergoing retinal imaging that have DR. Ultrasound-based imaging modalities are the most convenient modalities for carotid imaging. For retinal imaging, optical coherence tomography and angiography proved to be revolutionary in ophthalmology [263]. However, the use of fundus imaging is widely followed, with further possibilities explored using smartphone-based retinal imaging modalities. Further, for risk assessment, artificial intelligence-based algorithms are the most highly recommended. It is, therefore, recommended that all these factors be followed to identify and treat the disease at its earliest.

### 7.3. A Special Note on DR and Monitoring of CVD Risk

With our current understanding of DR and the existing interventions associated with it, we believe that the link between DR and CVD is not well established in the general population. This is the reason for the worsening statistics of both diseases. It is thus important to educate DR patients about their risk of CVD and provide the necessary monitoring options. It is necessary to provide this pipeline as an intervention in healthcare systems. With the help of robust imaging and monitoring techniques, several lives can be saved, and disease can be treated.

### 7.4. The Effects of COVID-19 on DR Patients

This section shows how DR got affected during the COVID-19 period. The importance of DR during pandemic times has risen several folds. SARS-CoV-2 was seen as an important barrier to DR management and this has led to the suspension of primary healthcare services such as limited access to diabetes-related medical consultations and retinal screenings [237]. Advocacy with the government is seen to be critical to facilitating continuous sustainable services as many DR screening services and referral initiatives have been adversely impacted by the COVID-19 pandemic [238]. Please note that, in the presence of diabetes, due to the aggravation of underlying pulmonary microcirculatory impairments, it has been observed that the underlying microvascular disease is associated with a higher risk of adverse DR outcomes in the COVID-19 period [239].

### 7.5. Strengths, Weaknesses, and Future Extensions

By introducing this link, we provide additional support to existing healthcare systems. It is rightly said that prevention is better than cure. With the knowledge about the link between DR and CVD as well as the low-cost screening using AI-based algorithms, not only can patients be treated, but disease can also be prevented from happening. One limitation that we feel is that there are no pipelines developed for treating DR patients for CVD and it is important to put more light on this aspect. With the ongoing pandemic, it is important to discuss how both the targeted diseases may be affected in the presence of the SARS-CoV-2 virus. In the future, we would like to discuss the role of big data in reducing the bias of cohort [264]. Further, we would focus on the role of different types of DL and ML-based classifiers like the ensemble machine learning (EML)-based algorithm and auto-encoders for automated DR detection [265,266] and carotid wall segmentation [221,262,263] and quantification [267,268,269,270,271,272,273,274].

## 8. Conclusions

This narrative review presented the role of diabetic retinopathy and its association with cardiovascular risks in diabetes patients. We also showed the complications of diabetes that lead to coronary heart disease. This review emphasized the hypothesis that DR can lead to the worsening of CVD. Thus, it is of utmost importance to identify CVD complications in DR patients. We also demonstrated that carotid imaging can be used as a low-cost, non-invasive alternative to the existing imaging modalities for CVD screening in DR patients. This low-cost B-mode ultrasound will also be beneficial for plaque tissue characterization in DR patients to provide an additional and vital understanding of the CVD risk. Further, we observed that AI-based solutions are powerful for risk stratification of CVD in DR patients. Lastly, we glance at the role of DR and CVD in the COVID-19 framework and observe how AI plays a role in this system.

## Figures and Tables

**Table 1:** The link between DR and CHD.

Citations	Year	PDR ^a^	CVD ^b^	RI ^c^	CHD ^d^	CI ^e^	AI ^f^	RS ^g^	DR-CVD Link	SOC ^h^
Hecke et al. [105]	2005	✓	✓	✕	✓	✕	✕	✕	✓	✓
Cheung et al. [110]	2007	✕	✓	✕	✓	✕	✕	✕	✓	✓
Kawasaki et al. [108]	2013	✓	✓	✕	✓	✕	✕	✓	✓	✓
Ellis et al. [109]	2013	✓	✓	✕	✓	✕	✕	✕	✓	✓
Pradeepa et al. [107]	2015	✕	✓	✓	✓	✕	✕	✕	✓	✓
Um et al. [111]	2015	✕	✓	✓	✓	✕	✕	✕	✓	✓
Barlovic et al. [103]	2018	✕	✓	✕	✓	✕	✕	✕	✓	✓
Xu et al. [112]	2020	✕	✓	✕	✓	✕	✕	✕	✓	✓

PDR ^a^: Pathophysiology of Diabetic Retinopathy, CVD ^b^: Cardiovascular Diseases, RI ^c^: Retinal Imaging, CHD ^d^: Coronary Heart Disease, CI ^e^: Carotid Imaging, AI ^f^: Artificial Intelligence, RS ^g^: Risk Stratification, SOC ^h^: Strength of Correlation.

**Table 2:** Difference between FI and OCT.

Modality	Image Formation	RF ^#^	Features of Interest	Limitations
FI	Colour photograph of the retinal surface.	7–20	Blood vessels, lesions, exudates, hemorrhages.	Dilation of pupils is often needed.
OCT	Near-infrared light penetrates the retina.	4	The internal retinal structure is shown in cross-section, including changes in the nerve fiber layer.	Susceptible to media opacities, does not visualize blood.

^#^: Resolution factor; FI: fundus imaging; OCT: optical coherence tomography.

**Table 3:** Studies showing evidence for the DR-CVD hypothesis.

SN	Author	Year	Imaging Device	Comorbidity	DR-CVD Link	Conclusion
1.	Liao et al. [128]	2004	Retinal imaging	hypertension, dyslipidemia, and diabetes mellitus	✓	Macro and microvascular disease support stroke prognosis.
2.	Minmoun et al. [123]	2009	Laser Doppler flowmetry	Retinal microvascular abnormalities	✓	retinopathy is correlated with white matter lesions in the brain and coronary calcification
3.	McClintic et al. [129]	2010	Retinal imaging	Type 2 diabetes	✓	Retinal vasculature abnormalities were related to coronary heart disease
4.	Liew et al. [130]	2010	Retinal imaging	CHD	✓	Fractal analysis on microvasculature predicted CHD mortality
5.	Freitas et al. [126]	2011	Color Doppler imaging	CHF	✓	Abnormalities in the optic nerve head in the eyes were related to CHF
6.	Flammer et al. [124]	2012	Color Doppler imaging	dyslipidemia, DM, or systemic hypertension	✓	CVD was found to be associated with macular degeneration and impaired autoregulation in the eyes.
7.	Seidelmann et al. [125]	2016	Retinal vessel imaging	ASCVE or heart failure (HF)	✓	Reduction in retinal arterioles and enlargement of retinal venules showed stroke and CHD
8.	Naegele et al. [127]	2017	Dynamic Retinal Vessel Analyzer	Smoking, hypertension, dyslipidemia, and diabetes mellitus	✓	In patients with CHF, the responsiveness of the retinal microvascular dilatation to flickering light was reduced.

**Table 4:** CVD risk stratification thresholds for statin initiation.

Guidelines	Risk Score	Cut-Off with Statin Initiation
ACC/AHA 2013 [175]	Risk Score for Pooled Cohorts	7.5% cutoff for starting a moderate to high-intensity statin
NICE 2014 [176,177,178]	QRISK2 risk engine	Offers atorvastatin 20mg daily who have a score ≥10%
Canadian 2012 [179]	FRS cardiovascular disease risk score	Offers atorvastatin 20mg daily a score of 10%
U.S. Preventive Services Task Force [180]	Risk Score for Pooled Cohorts	Low-to-Moderate Statin Dose in Risk > 10%

**Table 5:** Comparing the proposed review against previous reviews on joint DR and CVD.

Citations	Year	DR ^a^	CVD ^b^	RI ^c^	CI ^d^	AI ^e^	RS ^f^	COV-19 ^g^
Son et al. [22]	2010	✓	✓	✕	✓	✓	✓	✕
Alonso et al. [37]	2015	✓	✓	✕	✕	✕	✕	✕
Ting et al. [26]	2019	✓	✓	✓	✓	✓	✓	✕
Simó et al. [38]	2019	✓	✓	✕	✓	✕	✕	✕
Gupta et al. [39]	2021	✓	✕	✓	✕	✓	✕	✕
Proposed Review	2022	✓	✓	✓	✓	✓	✓	✓

DR ^a^: Diabetic Retinopathy: CVD ^b^: Cardiovascular Diseases, RI ^c^: Retinal Imaging, CI ^d^: Carotid Imaging AI ^e^: Artificial Intelligence, RS ^f^: Risk Stratification, COV-19 ^g^: COVID-19.

## Data Availability

No data availability.

## Abbreviations

ACC	American College of Cardiology
AECRS	AtheroEdge Composite Risk Score
AGE	Advance Glycation End Products
AHA	American Heart Association
AHEAD	Action for Health in Diabetes
AI	Artificial Intelligence
ASCVD	Atherosclerotic Cardiovascular Disease
CAD	Coronary Artery Disease
CAPB	Coronary Artery Plaque Burden
CCA	Common Carotid Artery
CHF	Congestive Heart Failure
CI	Confidence Interval
cIMT	Carotid Intima-Media Thickness
CT	Computerized Tomography
CVA	Cerebrovascular Accident
CVD	Cardiovascular disease
CVE	Cardiovascular Events
CCVRC	Conventional Cardiovascular Risk Calculator
DL	Deep Learning
DM	Diabetes Mellitus
DME	Diabetic Macular Edema
DR	Diabetic Retinopathy
EML	Ensemble Machine Learning
FA	Fluorescein Angiography
FRS	Framingham Risk Score
GLS	Global Longitudinal Strain
HR	Hazard Ratio
ICA	Internal carotid artery
ICGA	Indocyanine Green Angiography
IMTV	Intima-Media Thickness Variability
IVUS	Intravascular Ultrasound
MA	Macular Edema
MI	Myocardial Infarction
ML	Machine Learning
MRI	Magnetic Resonance Imaging
MVD	Microvascular Disease
NICE	National Institute for Health and Care Excellence
NPDR	Non-Proliferative Diabetic Retinopathy
OCT	Optical Coherence Tomography
OpA	Ophthalmic artery
OR	Odds Ratio
PCRE	Pooled cohort risk equation
PDR	Proliferative Diabetic Retinopathy
PET	Positron Emission Tomography
PKC	Protein Kinase C
PVD	Peripheral Vascular Disease
RRS	Reynold Risk Score
T2DM	Type 2 Diabetes Mellitus
TIR	Time in Range
UKPDS	UK Prospective Diabetes Score
US	Ultrasound
UTC	Ultrasound-based Tissue characterization
COVID-19	Coronavirus-2019
WHO	World Health Organization
